# Towards New Approaches to Evaluate Dynamic Mosaicism in Ring Chromosome 13 Syndrome

**DOI:** 10.1155/2019/7250838

**Published:** 2019-12-28

**Authors:** Cristian Petter, Lilia Maria Azevedo Moreira, Mariluce Riegel

**Affiliations:** ^1^SARAH Network of Rehabilitation Hospitals, Salvador, BA, Brazil; ^2^Post Graduate Program in Genetics and Biodiversity, Universidade Federal da Bahia, Salvador, BA, Brazil; ^3^Genetics & Society Program, Universidade Federal da Bahia, Salvador, BA, Brazil; ^4^Medical Genetics Service, Hospital de Clínicas de Porto Alegre, Porto Alegre, RS, Brazil; ^5^Post Graduate Program in Genetics and Molecular Biology, Universidade Federal do Rio Grande do Sul, Porto Alegre, RS, Brazil

## Abstract

Individuals with ring chromosome 13 may show characteristics observed in a deletion syndrome and could present a set of dismorphies along with intellectual disability, according to chromosomal segments involved in the genetic imbalance. Nevertheless, ring anomalies likewise is called “dynamic mosaicism”, phenomena triggered by the inner instability concerning the ring structure, thus leading to the establishment of different cell clones with secondary aberrations. Phenotypic features, such as growth failure and other anomalies in patients with this condition have been associated with an inherent ring chromosome mitotic instability, while recent studies offer evidence on a role played by the differential loss of genes implicated in development. Here, we observed similar mosaicism rates and specific gene loss profile among three individuals with ring chromosome 13 using GTW-banding karyotype analyses along with FISH and CGH-array approaches. Karyotypes results were: patient 1—r(13)(p13q32.3), patient 2—r(13)(p11q33.3), and patient 3—r(13)(p12q31.1). Array-CGH has revealed qualitative genetic differences among patients in this study and it was elusive in precise chromosomal loss statement, ranging from 13 Mb, 6.8 Mb, and 30 Mb in size. MIR17HG and ZIC2 loss was observed in a patient with digital anomalies, severe growth failure, microcephaly and corpus callosum agenesis while hemizygotic EFNB2 gene loss was identified in two patients, one of them with microphtalmia. According to these findings, it can be concluded that specific hemizygotic loss of genes related to development, more than dynamic mosaicism, may be causative of congenital anomalies shown in patients with ring 13 chromosome.

## 1. Introduction

Ring chromosomes originate from the break and rejoining of both chromosome arms and consequently formation of a circular rearrangement, most often with a genetic loss of the extremities. Ring Chromosome 13 is observed in around 20% of ring cases in still-births and its prevalence is estimated in 1/58,000 births [1, 2]. Clinical findings seem associated with the size of the rings, concerning the extent of deleted segments, but also on the ring instability, which results in more severe features [3–9]. Phenotype usually consists of anencephaly, aprosencephaly or encephalocele along multiple and more severe congenital anomalies in minor rings (larger deletions) involving bands 13q14 and/or q22. Larger ring 13 cases (smaller deletions) show milder phenotypic implications including ophthalmic and extremities malformations and a prolonged life expectancy. In cases with even larger rings, individuals may not present any dismorphic traits, although a mental deficiency would be detected [10]. Clinical characteristics observed in ring 13 individuals usually overlap with linear 13q deletions, although more severe traits are commonly diagnosed because of the mitotic instability of the ring.

Some authors attribute the phenotypic features seen in ring 13 patients to a “ring chromosome syndrome” through a mechanism named “dynamic mosaicism” as postulated by Kosztolanyi [11], while recent reports point to a loss of development genes during the ring formation as a main role in the process.

Studies of genotype-phenotype correlation delineate the 13q- syndrome and thus provide an effective understanding of that clinical entity [2, 12–17]. Identification of specific genes and their respective association to clinical traits has led, for instance, to the establishment of *ZIC2* as a hallmark in cases of 13q-, due to the fact that this gene loss is causative of holoprosencephaly [18]. Additional studies have been trying to associate a number of genes to the sort of clinical characteristics found in this syndrome, for instance, *EFNB2* to microphtalmia [13, 15] and *ARHGEF7* to mental retardation/microcephaly [19, 20].

Here we present three new cases of individuals with ring chromosome 13 and their characterization using banding cytogenetics and fluorescence in situ hybridization (FISH) analysis along with array-comparative genomic hybridization (array-CGH) approach in order to evaluate the rate of cell mosaicism in each individual and likewise to ascertain differences in genetic profile on the genotype-phenotype correlation.

## 2. Materials and Methods

**Figure 1:**
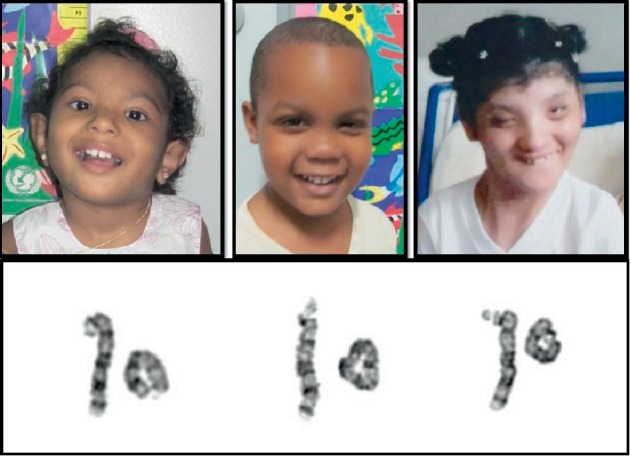
Left to right, patients 1, 2, and 3 and their respective ring chromosome 13 (bottom).

Patients previously referred to genetic counseling and diagnosed with ring chromosome 13, were reevaluated in the Genetics and Society Program–Biology Institute/Universidade Federal da Bahia (UFBA), Salvador, Brazil (Figure 1). Peripheral blood samples for GTW-banding and FISH karyotype, and aCGH were obtained from probands after informed consent.

## 3. Casuistics

### 3.1. Case 1

The patient was born on October 04, 2012, female, healthy parents with normal karyotype. At birth, the mother was 24-years-old, G2/P1/A0, and reported infrequent fetal movements during pregnancy, beginning in the 4^th^month. Pregnancy was not accompanied by any medical care. The delivery was normal, with Apgar 8/9 at 1 and 5 minutes. The birth weight (1600 g) and height (42 cm) were below the 3^rd^ percentile and microcephaly, floated nasal bridge, large ears and facial dysmorphysm were observed. In the 4^th^month a transfontanellar ultrasound showed mild ectasy of the right lateral ventricle corpus and a ventricular parenchymal increase. The patient showed global development delay and sat only with support in about the 8^th^month. Anterior fontanel closed between the 9^th^ and 17^th^ month. Serological tests were negative for Cytomegalovirus and Toxoplasma; inborn errors of metabolism tests were normal. After the first year, OFC was 35.5 cm (below 2^nd^ percentile) and the child could not roll, crawl or walk without support.

### 3.2. Case 2

The patient was born on March 26, 2008, male, healthy parents with normal karyotype. The birth weight (2620 g) and height (48 cm) were within the 3^rd^ percentile. At 4½ years old, OFC was 47.5 cm (below the 2^nd^ percentile). The mother reported that there were no complications during pregnancy, with cesarean delivery at term. The child had a weak cry, achieved cephalic equilibrium in a few months and lordosis was observed. The child walked after being two-years-old. Electroencephalogram was normal. Currently, the child is attentive and participatory, showing microcephaly, hypotonia, epicanthus, floated nasal bridge, high ogival palate, short neck, with small hands and feet.

### 3.3. Case 3

The patient was born on October 1^st^, 1989, female, healthy parents with normal karyotype. The birth weight was 1200 g (below 3^rd^ percentile) while the height was not registered. The mother was 28 and the father was 21-years-old. Fetal movements were evident in the 4^th^ month. Delivery was normal and premature (approximately 24 weeks), with amniotic fluid loss and suspicion of anoxya. The child remained in the ICU for 15 days and 4 days in the nursery. TORCH test was negative. Upon physical examination, microcephaly, hypertelorism, right thumb agenesis, heart murmur, dyspnea, and neonatal jaundice were observed. CT revealed corpus callosum agenesis. At 4 months, the patient weighed 4100 g and her height was 53 cm (both measurements below 3^rd^ percentile) and OFC was 33 cm (below 2^nd^ percentile). Currently, the proband presented cognitive disability, global development delay, hypotonia, absence of speech, and multiple dysmorphisms. Currently, she is completely dependent for daily life activities, and spends all the time in the sitting or lying position.

#### 3.3.1. Karyotype Analysis

A total of 500 metaphases from each patient were attained by means of GTW-banding after 96 hours of peripheral blood cell cultive in RPMI 1640 medium and thymidine (both GibCo, USA) and synchronization following standard procedures [21]. Chromosomal analysis was performed in a band resolution range average of 400–700 (GTW) [22].

#### 3.3.2. Fluorescence In Situ Hybridization (FISH)

FISH studies were performed according to manufacturer protocols (Cytocell, UK) using whole chromosome paint 13 probe (WCP13) and Rb1/Tel13q probe in order to characterize the origin of either ring or marker chromosomes observed in banding cytogenetics examinations. A total of 150 cells were addressed for each probe for all the patients in this study.

#### 3.3.3. Array-CGH

Whole-genome analysis was performed by means of the Agilent Human Genome CGH microarray 60-mer Oligonucleotide-based microarray (8 × 60 K, Agilent Technologies Inc., Santa Clara, CA) with a 40 kb resolution. Labeling and hybridization were performed according to the manufacturer protocols and analyses were made through the microarray scanner (G2600D) and the Feature Extraction software (v9.5.1) (Agilent Technologies). Image analyses were created by means of Agilent Genomic Workbench Lite Edition 6.5.0.18 along with the statistical algorithm ADM-2 and sensitivity threshold 6.0.

## 4. Results

The patients described here showed a remarkable variation in phenotypes, with severity of clinical findings consistently being related to the extension of the deleted segments. Clinical data of them may be assessed in Table 1.

All individuals presented cognitive disability although with distinctive levels. Patient 2 (6.87 Mb deletion) showed milder clinical findings, while patient 3 (30 Mb deletion) was more affected and patient 1 (13.58 Mb deletion) apparently had an intermediate phenotype. Cases 1 and 3, which presented the largest deletions, additionally shared the following characteristics: low birth weight, microcephaly, facial dysmorphisms, oblique eyelids, and dysmorphic ears. The case 3 was the most clinically severe amongst the group, with brain anomalies, low-set hair, hirsutism, nistagmus, strabism, micrognathism. microstomy, mammilary hypertelorism, feet anomalies and thumb hypoplasia/agenesis. Interestingly, hands and skeletal anomalies along with congenital cardiopathy were observed only in patients 2 and 3 where the smaller and the larger deletions were detected.

### 4.1. Cytogenetics

GTG-banding analysis (adapted from [23]) was performed in order to determine the karyotype and also to verify the occurrence of low-rate cell mosaicism. Metaphases was obtained after a 72 h lymphoblastoid cell cultures (adapted from [21]). A total of 500 metaphases for each proband was counted and carefully identificated, following the documentation of representative chromosome aberrations. In all cases, the main cell lineage (84.2–88.8%) was composed of the monocentric ring, followed by 13 monosomy and subsequently by a number of secondary rearrangements Table 2. A normal cell clone, not previously identificated, was observed in patient 3.

### 4.2. Fluorescence In Situ Hybridization (FISH)

**Figure 2:**
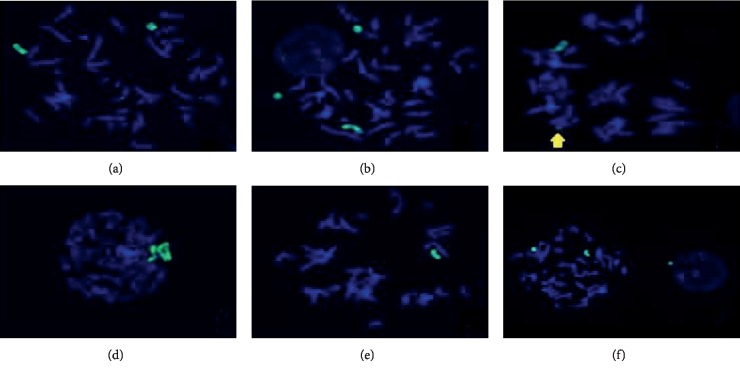
WCP13 probe (green): (a) patient 2, normal 13 and monocentric ring; (b) patient 1, two monocentric rings and normal 13; (c) patient 3, normal 13 and marker with positive hybridization (arrow); (d) patient 2, normal 13 and dicentric ring; (e) patient 1, single normal 13 in a monosomic cell; (f) patient 1, on the left, metaphase with monocentric ring and normal 13, near an interphasic nucleus bordered by a micronuclei, the latter also showing positive hybridization.

**Figure 3:**
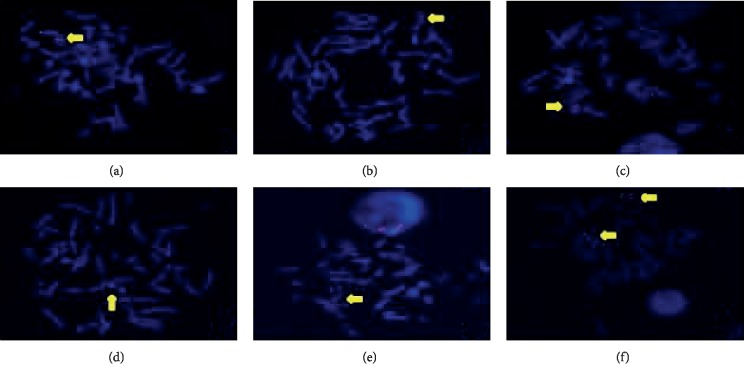
Rb1 (red)/Tel13q (green) probes: (a) patient 1, monocentric ring showing Rb1 signal and no Tel13q; (b) patient 2, single normal 13 in a monosomic cell; (c) patient 3, dicentric ring presenting double Rb1 signal; (d) Patient 2, marker chromosome without both Rb1 and Tel13q signals; (e) Patient 2, two dicentric rings with double Rb1 signal each; (f) Patient 3, normal cell.

The FISH WCP13 probe allowed for the identification of the origin of both ring and marker chromosomes observed in GTW-banding cytogenetics examination. Micronuclei were likewise noted in all patients, and the use of WCP13 permitted the assessment of the chromosome 13 origin of such structures Figure 2(f). The Rb1/Tel13q probe indicated deletion of subtelomere 13q in the whole ring chromosomes of probands in this study. Otherwise, Rb1 structural deletion was observed in very small ring chromosomes (markers). Nevertheless, the presence of a normal karyotype cell clone in patient 3 was also confirmed Figure 3(f).

### 4.3. Array-Comparative Genomic Hybridization

**Figure 4:**
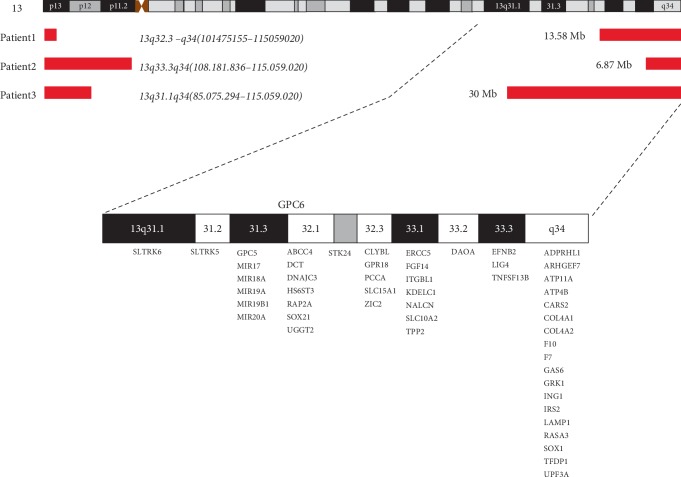
Schematic distribution of deleted genes and sizes of deletions of probands (red bars). Order of appearance of genes in the bands does not necessarily reflect the actual sequence in the chromosome (ideogram from above extracted from UCSC Genome Browser Gateway).

**Figure 5:**
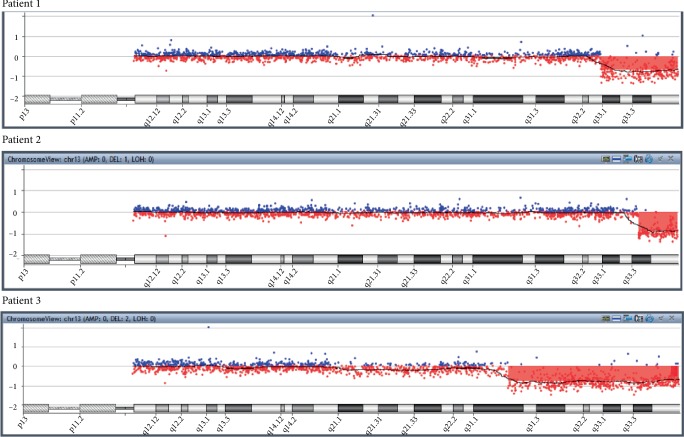
aCGH profile indicating the segment deletions on chromosome 13q.

All individuals showed a terminal deletion in 13q with genes losses Table 3. Patient 1 revealed a 13.58 Mb loss affecting bands q32.3–q34 between genomic positions 101475155–115059020. Patient 2 presented a 6.87 Mb deletion affecting bands q33.3–q34 between genomic positions 108181836–115059020. Patient 3 showed a deletion of 30 Mb affecting bands q31.1–q34 between genomic positions 85.075.294–115.059.020 *(UCSC Genome Browser on Human Feb. 2009 (grhH37/hg19)* (Figures 4 and 5).

## 5. Discussion

In this study, classical cytogenetic tools were combined with biomolecular approaches in order to assess the cell mosaicism in individuals with ring chromosome 13 and to perform genotype-phenotype association to the 13q- syndrome. Brown et al. [24] describe three deletion groups in 13q: group 1, deletions proximal to q32, group 2, breakpoints within q32, and group 3, deletions of q33 and q34. Patients 1—r(13)(p13q32.3)—and 3—r(13)(p12q31.1) were classified as deletion group 2, while patient 2—r(13)(p11q33.3) was compatible with group 3.

FISH analysis with WCP13 confirmed the chromosome 13 origin of small markers, large dicentric rings, and even pulverized material. Interestingly, micronuclei eventually observed in cytogenetics examination showed positive WCP13 hybridization, indicating the loss of ring chromosomes. In fact, Ford et al. [25] inferred that the majority or even the totality of micronuclei would correspond to either whole chromosomes or chromatids due to mitotic arrestment. On the other hand, Rb1/Tel13q- indicated deletion of subtelomeric 13q sequences in all ring chromosomes as well as in their derivatives. Hemizygotic Rb1 deletion was revealed in very small marker chromosomes at a low incidence.

The low-rate mosaicisms observed in cytogenetic examination were not detected by a-CGH, as described elsewhere [2, 8, 26–28]. According to Liehr et al. [29], cytogenetics is still considered as the gold-standard test for mosaicism identification while the refinement of aberrations should be performed by cytomolecular approaches. In fact, a-CGH was a determinant to accurately address the size of deletions in probands. Data obtained directly correlated to the severity of patient's clinical findings, as reported elsewhere [2, 13, 15–17].

GTW-banding such as FISH analysis in a comprehensive number of cells for each patient did not reveal significant differences in the frequencies of cell clones among individuals, despite the unequal size of deletions observed in the group, as referred to by Sodre et al. [7]. This is in disagreement with Kosztolanyi [11] postulated, who pointed to a correlation between ring size and ring instability. In each proband, the clonal distributions were very close and showed the same proportion reported elsewhere in cases of ring chromosomes [1, 2, 7, 28, 30–34].

Kosztolanyi [31] attributes the mitotic ring instability to a unique entity called “ring syndrome”, irrespective of the chromosomal origin, whose characteristics would include extreme growth failure, few or no minor anomalies and moderate to high mental retardation. The phenotypic characteristics would be due to the apoptosis of nonviable cells with secondary aberrations and thus lead to growth failure. However, Sodre et al. [7] pointed out that cells derived from secondary ring aberrations could multiply and survive *in vivo*, and so contribute to the phenotypic variations observed in patients with ring chromosomes, in opposition to the apoptotic mechanism of Kosztolanyi [31].

Further, Rossi et al. [5, 6] pointed out evidence of specific losses of genetic elements to be the real cause of short stature in ring patients. The authors refer to a group of ring 15 patients whose probands with short stature presented deletion of IGF1R (insulin-like growth factor 1 receptor precursor). Additionally, Glass et al. [35] reported the same results in another cohort of ring chromosome 15 individuals. Strong evidence in the latter study against the theory of a ring chromosome syndrome was the fact that, in the majority of cases, a cryptic deletion was the current cause of the phenotypic anomalies.

More recents evaluations by the means of aCGH have allowed the identification of a number of genes in deleted chromosome regions. However, only few reports postulate an improvement of a genotype-phenotype correlation with this new approach [2, 9, 12–16, 27, 28, 36–39].

Concerning 13q- haploinsufficiency, Amor et al. [40] described a boy with growth hormone deficiency and hand anomalies which presented a neocentric ring 13q31–32. The authors suggested three candidate genes that might be contributing to the boy's phenotype, *GPC5* and *FARP1* for the hand anomalies and *SOX21* for the short stature. Other authors also suggested the association of GPC5 with those anomalies [14, 15, 36]. However, recent evidence shown by DePontual et al. [39] attributes this to the miRNA cluster *MIR17HG*, and not to *GPC5*, a causative factor in the syndrome of growth failure, hand anomalies, and microcephaly. According to the authors, this syndrome would be the first example of a miRNA gene responsible for a syndromic development defect in humans. Our patient 3 showed short stature, microcephaly, and hand anomalies with thumbs aplasia/hypoplasia and the patient lacks the *MIR17HG* cluster. Nevertheless, one of the DePontual's patients also presented hypoplasic thumb. Kirchhoff et al. [14] referred to two patients with thumb a-/hypoplasia and indicated a separate entity within the 13q31.3q33.1 segment. It seems essential to carry out new investigations aimed at the miRNAs as possible causes of novel human syndromes, as according to DePontual et al. [39], new evidence has been emerging about the miRNAs in development modulation.

The *ZIC2* gene is considered a hallmark in 13q- syndrome as it is implicated with brain anomalies [13–15, 18, 41]. Patient 3 showed hemizygotic loss of this gene and presented brain anomalies. Patient 1, despite belonging to deletion group 2, involving band 13q32.3 where *ZIC2* is located, did not show brain anomalies and presented both alleles of this gene.

An-/microphthalmia, a condition which has been linked to both the EFNB2 gene [13, 15] and the SOX1 gene [14] was noted only in individual 3, although both patients 1 and 3 showed deletion of *EFNB2* and the three cases revealed haploinsufficiency of *SOX1*. Thus, our data weakens an association of both genes to eye malformations. On the other hand, *SOX1* has also been implicated in microcephaly and cortical malformations [14, 19] together with *ARHGEF7*, deleted in our group [14, 19, 20]. Microcephaly was a condition observed in all probands in this study and is a common finding in patients with 13q deletions [13, 14, 24]. A minimal critical deletion for this trait was determined for 13q34 [2, 17].

Although a gene could apparently lack a causative link to a phenotypic trait, one cannot exclude its involvement in that feature. Another mechanism proposed to explain the clinical anomalies in deletions would be the “unmasking” of recessive genes lying in the normal homologue chromosome through the loss of chromosomal segments in the rearrangement [5, 6, 42]. The emergence of a trait, in this case, would depend on the alleles lying in the normal homologue.

In this study we observed the presence, though in very low frequency, of marker chromosomes with deletion of Rb1. One knows that the occurrence of constitutive mutation at one of the Rb1 alleles is implicated in an estimated risk of 400x in retinoblastoma and other malignancies [43]. Despite the fact that a cytogenetic loss of Rb1 would not necessarily lead to the development of neoplasies [44], rare cases of ring chromosome 13 related to retinoblastoma were reported [45].

This study agree with the point of view of Rossi et al. [5, 6] that phenotypic features in ring cases depend mainly on the size of deleted segments and respective loss of development genes, although the effect of the ring instability could not be excluded. However additional investigations to clarify this phenomenon are yet recommended.

## 6. Conclusions

We believe the combination of multiple cytogenetics and cytomolecular approaches to be critical for the genetic evaluation of syndromic and nonsyndromic individuals with ring chromosome 13 syndrome.

The Array-CGH approach is currently an indispensable tool for the evaluation of cryptical aberrations as well as for performing genotype-phenotype studies. However, classical cytogenetics strategies are still the methods of choice in the investigation of either cell mosaicism or balanced rearrangements.

Additional genotype-phenotype studies are needed in order to associate genes to their theoretical functions, thus providing a guide for further genomic research.

Our data indicates that the deletion of development genes may be the main cause of phenotypic variation, at least in cases of ring chromosome 13.

## Figures and Tables

**Table 1:** Clinical and general findings of patients evaluated.

Patients	1	2	3
DOB (d/m/y)	04/10/2012	26/03/2008	01/10/1989
Euchromatic deleted segment	q32.3q34	q33.3q34	q31.1q34
Segment size	13.58 Mb	6.87 Mb	30 Mb
Sex	F	M	F
Weight at birth (g)	1600	2620	1200
Height at birth (cm)	42	48	NK

Microcephaly	+	+	+
Mental retardation	+	+	+
Brain anomalies	−	−	+
Development delay	+	+	+
Hypotonia	+	+	+
Facial dysmorphisms	+	−	+
Prominent forehead	−	+	−
Low-set hair	−	−	+
Hirsutism	−	−	+
Broad/flat nasal bridge	−	+	−
Oblique eyelids	+	−	+
Microphthalmia	−	−	+
Nistagmus	−	−	+
Strabismus	−	−	+
Epicanthus	+	+	−
Hypertelorism	+	−	−
Micrognathism	−	−	+
Microstomy	−	−	+
Narrow and ogival palate	−	+	Nk
Dysmorphic ears	+	−	+
Low ears implantation	+	−	+
Short neck	−	+	−
Mammilary hypertelorism	−	−	+
Congenital cardiopathy	−	+	+
Skeletal anomalies	−	+mild lordosis	+
Feet anomalies	−	−	+
Hands anomalies	−	+	+
Thumb hypoplasia/agenesis	−	−	+
Parental karyotypes	Normal	Normal	Normal

*Notes.* nk: not known (+): presence (‒): absence

**Table 2:** Absolute and relative frequencies of clonal and nonclonal cytogenetic findings and distribution in probands (500 metaphases/individual).

Cell lineage	Karyotype	Patient 1	Patient 2	Patient 3
Normal	46,X_	—	—	10 (2.0%)
Monocentric ring	46,X_,r(13)	421 (84.2%)	429 (85.8%)	421 (84.2%)
Monosomy 13	45,X_,‒13	59 (11.8%)	40 (8.0%)	46 (9.2%)
Dicentric ring	46,X_,dic r(13)	3 (0.6%)	21 (4.2%)	5 (1.0%)
Monosomy 13 + marker	46,X_,‒13,+mar	3 (0.6%)	3 (0.6%)	6 (1.2%)
Deletion 13?	46,X_,?del(13)(q31)	1 (0.2%)	—	—
Isochromosome 13q	46,X_,i(13)(q10)	—	1 (0.2%)	—
Derivative13	46,X_,der(13)	4 (0.8%)	—	—
Monocentric ring × 2	47,X_,r(13) × 2	4 (0.8%)	1 (0.2%)	4 (0.8%)
Dicentric ring × 2	47,X_,dic r(13) × 2	2 (0.4%)	1 (0.2%)	1 (0.2%)
Monocentric ring + dicentric ring	47,X_,r(13),+dic r(13)	1 (0.2%)	2 (0.4%)	1 (0.2%)
Monocentric ring + derivative 13	47,X_,r(13),+der(13)	1 (0.2%)	—	—
Monocentric ring + marker	47,X_,r(13),+mar	1 (0.2%)	1 (0.2%)	1 (0.2%)
Dicentric ring + marker 1 + marker 2	49,X_,dic r(13),+mar1 × 2,+mar2	—	1 (0.2%)	—
Pulverization	46,X_,pvz(13)	—	—	3 (0.6%)
Monocentric ring + pulverization	47,X_,r(13),+pvz(13)	—	—	2 (0.4%)
N=		500	500	500

**Table 3:** List of deleted genes^∗^ in probands.

Gene	OMIM	Description	Patient 1	Patient 2	Patient 3
*ABCC4*	605250	ATP-binding cassette, subfamily C, member 4			^∗^
*ADPRHL1*	610620	ADP-ribosylhydrolase 1	^∗^	^∗^	^∗^
*ARHGEF7*	605477	Rho guanine nucleotide exchange factor 7	^∗^	^∗^	^∗^
*ATP11A*	605868	ATPase, class VI, type 11A	^∗^	^∗^	^∗^
*ATP4B*	137217	ATPase, H^+^, K^+^ transporting, beta	^∗^	^∗^	^∗^
*CARS2*	612800	Cysteinyl-tRNA synthetase 2	^∗^	^∗^	^∗^
*CLYBL*	609686	Citrate lyase beta-like			^∗^
*COL4A1*	120130	Angiopathy, hereditary, with nephropathy, aneurysmal	^∗^	^∗^	^∗^
*COL4A2*	120090	Collagen IV, alpha-2 polypeptide	^∗^	^∗^	^∗^
*DAOA*	607408	{Schizophrenia}, 181500 (2)	^∗^		^∗^
*DCT*	191275	Dopachrome tautomerase			^∗^
*DNAJC3*	601184	DNA J, E. coli, homolog of, subfamily C, member 3			^∗^
*EFNB2*	600527	Eph-related receptor tyrosine kinase ligand 5	^∗^		^∗^
*ERCC5*	133530	Cerebrooculofacioskeletal syndrome 3 (3)	^∗^		^∗^
*F10*	227600	Factor X deficiency (3)	^∗^	^∗^	^∗^
*F7*	227500	Factor VII deficiency (3)	^∗^	^∗^	^∗^
*FGF14M*	601515	Spinocerebellar ataxia-27, 609307(3)	^∗^		^∗^
*GAS6*	600441	Growth arrest- specific 6	^∗^	^∗^	^∗^
*GPC5*	602446	Glypican 5			^∗^
*GPC6*	604404	Glypican 6			^∗^
*GPR18*	602042	G protein-coupled receptor-18			^∗^
*GRK1*	180381	Oguchi disease-2, 258100 (3)	^∗^	^∗^	^∗^
*HS6ST3*	609401	Heparan sulfate 6-O-sulfotransferase 3			^∗^
*ING1*	601566	Squamous cell carcinoma, head and neck 275355	^∗^	^∗^	^∗^
*IRS2*	600797	{Diabetes mellitus, noninsulin-dependent} 125853	^∗^	^∗^	^∗^
*ITGBL1*	604234	Integrin, beta-like 1	^∗^		^∗^
*KDELC1*	611613	KDEL motif-containing 1	^∗^		^∗^
*LAMP1*	153330	Lysosome-associate membrane protein-1	^∗^	^∗^	^∗^
*LIG4*	601837	LIG4 syndrome, 606593 (3)	^∗^	^∗^	^∗^
*MIR17*	609416	Micro RNA 17			^∗^
*MIR18A*	609417	Micro RNA 18A			^∗^
*MIR19A*	609418	Micro RNA 19A			^∗^
*MIR19B1*	609419	Micro RNA 19B1			^∗^
*MIR20A*	609420	Micro RNA 20A			^∗^
*NALCN*	611549	Sodium leak channel, nonselective	^∗^		^∗^
*PCCA*	232000	Propionicacidemia, 606054 (3)			^∗^
*RAP2A*	179540	RAP2, member of RAS oncogene superfamily (K-rev)			^∗^
*RASA3*	605182	Ras p21 protein activator 3	^∗^	^∗^	^∗^
*SLC10A2*	601295	Bile acid malabsorption, primary (3)	^∗^		^∗^
*SLC15A1*	600544	Solute carrier family 15 (oligopeptide transporter)			^∗^
*SLTRK5*	609680	SLIT- and NTRK-like family, member 5			^∗^
*SLTRK6*	609681	SLIT- and NTRK-like family, member 6			^∗^
*SOX1*	602148	SRY (Sex determining region Y) box-1	^∗^	^∗^	^∗^
*SOX21*	604974	SRY (Sex determining region Y) box-21			^∗^
*STK24*	604984	Serine/Threonine protein kinase 24			^∗^
*TFDP1*	189902	Transcription factor Dp-1	^∗^	^∗^	^∗^
*TNFSF13B*	603969	Tumor necrosis factor ligand superfamily, member 13B	^∗^	^∗^	^∗^
*TPP2*	190470	Tripeptidyl peptidase II	^∗^		^∗^
*UGGT2*	605898	UDP-glucose glycoprotein glucosyltransferase 2			^∗^
*UPF3A*	605530	UPF3 regulator of nonsense transcripts	^∗^	^∗^	^∗^
*ZIC2*	603073	Holoprosencephaly-5, 609637 (3)			^∗^
